# Synergistic effects of exercise, cognitive training and vitamin D on gait performance and falls in mild cognitive impairment—secondary outcomes from the SYNERGIC trial

**DOI:** 10.1093/ageing/afaf242

**Published:** 2025-09-12

**Authors:** Frederico Pieruccini-Faria, Surim Son, Guangyong Zou, Quincy J Almeida, Laura E Middleton, Nick W Bray, Maxime Lussier, J Kevin Shoemaker, Mark Speechley, Teresa Liu-Ambrose, Amer M Burhan, Richard Camicioli, Karen Z H Li, Sarah Fraser, Nicolas Berryman, Louis Bherer, Manuel Montero-Odasso

**Affiliations:** Department of Medicine, Division of Geriatric Medicine, Schulich School of Medicine and Dentistry, Western University, London, Ontario, Canada; Parkwood Institute, Gait and Brain Laboratory, London, Ontario, Canada; Parkwood Institute, Gait and Brain Laboratory, London, Ontario, Canada; Western University, Schulich School of Medicine and Dentistry, Department of Epidemiology and Biostatistics, London, Ontario, Canada; Western University, Schulich School of Medicine and Dentistry, Department of Epidemiology and Biostatistics, London, Ontario, Canada; Alimentiv Inc., London, Ontario, Canada; Carespace Health and Wellness Neurodegeneration Clinics, Waterloo, Ontario, Canada; Department of Kinesiology and Health Sciences, University of Waterloo, Waterloo, Ontario, Canada; Schlegel-UW Research Institute for Aging, Waterloo, Ontario, Canada; Parkwood Institute, Gait and Brain Laboratory, London, Ontario, Canada; Recovery and Performance Laboratory, Biomedical Sciences, Faculty of Medicine, Memorial University of Newfoundland, St. John’s, Newfoundland and Labrador, Canada; Centre de Recherche de l’Institut Universitaire de Gériatrie de Montréal, Université de Montréal, Montreal, Quebec, Canada; École de Réadaptation, Faculté de Médecine, Université de Montréal, Montreal, Québec, Canada; School of Kinesiology, University of Western Ontario, London, Ontario, Canada; Department of Epidemiology and Biostatistics, and Schulich Interfaculty Program in Public Health, Western University, London, Ontario, Canada; Department of Physical Therapy, University of British Columbia, Vancouver, British Columbia, Canada; Djavad Mowafaghian Centre for Brain Health, Vancouver Coastal Health Research Institute, Vancouver, British Columbia, Canada; Centre for Aging SMART at Vancouver Coastal Health, Vancouver Coastal Health Research Institute, Vancouver, British Columbia, Canada; Ontario Shores Centre for Mental Health Sciences, Whitby, Ontario, Canada; Department of Psychiatry, Temerity Faculty of Medicine, University of Toronto, Toronto, Ontario, Canada; Department of Medicine and Neuroscience and Mental Health Institute, University of Alberta, Edmonton, Alberta, Canada; Department of Psychology, Concordia University, Montréal, Québec, Canada; Interdisciplinary School of Health Sciences, University of Ottawa, Ottawa, Ontario, Canada; Département des Sciences de l’Activité Physique, Université du Québec à Montréal, Montréal, Québec, Canada; Institut national du sport du Québec, Montréal, Québec, Canada; Centre de Recherche de l’Institut Universitaire de Gériatrie de Montréal, Montréal, Québec, Canada; Centre de Recherche de l’Institut Universitaire de Gériatrie de Montréal, Montréal, Québec, Canada; Centre de Recherche, Institut de Cardiologie de Montréal, Montréal, Québec, Canada; Département de Médecine, Université de Montréal, Montréal, Québec, Canada; Department of Medicine, Division of Geriatric Medicine, Schulich School of Medicine and Dentistry, Western University, London, Ontario, Canada; Parkwood Institute, Gait and Brain Laboratory, London, Ontario, Canada; Western University, Schulich School of Medicine and Dentistry, Department of Epidemiology and Biostatistics, London, Ontario, Canada

**Keywords:** gait, exercises, cognitive, training, vitamin D, older people, Mild Cognitive Impairment, Falls

## Abstract

**Background:**

Older adults with mild cognitive impairment (MCI) have a higher risk of gait impairments and falls; yet, the effects of multimodal interventions, including combinations of exercises with cognitive training, on improving their mobility remain unclear.

**Objectives:**

To investigate the synergistic effects of aerobic-resistance exercise combined with cognitive training, with or without vitamin D supplementation, on gait performance and falls risk in older adults with MCI.

**Methods:**

The effect of 20 weeks of aerobic-resistance exercise, cognitive training, and Vitamin D supplementation (10 000 IU 3×/week) on gait and falls in older adults with MCI was evaluated in the SYNERGIC trial, using a fractional factorial design. Assessments were conducted at baseline, 6-month endpoint (after intervention) and 12-month endpoint (follow-up). Eligible participants were between the ages of 65 and 84 years with MCI enrolled from 19 September 2016 to 7 April 2020. Main outcomes of interest for gait performance were gait speed and gait variability changes, whilst for falls were incidental falls and incidental injurious falls.

**Results:**

Amongst 161 participants, the four exercise-based arms improved gait speed (+7.5 cm/s, *P* < .001) and reduced falls (incidence rate ratios (IRR) = 0.65, 95% confidence interval (CI): 0.32–1.42, *P* = .25) and injurious falls (IRR = 0.38, 95% CI: 0.15–1.05, *P* = .05) at 6-month endpoint. Falls reduction reached statistical significance (IRR = 0.28, 95% CI: 0.13–0.64, *P =* .002) at 12-month endpoint. Exercises combined with cognitive training showed the greatest gains in gait speed at 6-month endpoint (*P* < .001) and in reducing falls at 12-month endpoint (IRR = 0.24, 95% CI: 0.05–0.77, *P* = .02) compared to the control. Vitamin D did not enhance outcomes and increased gait variability, a marker of instability.

**Conclusion:**

Aerobic-resistance exercise combined with sequential computerised cognitive training improved gait performance at 6 months and decreased the risk of falls and injuries at 12 months in older adults with MCI. The addition of vitamin D did not produce benefits.

## Key Points

- Twenty weeks of combined aerobic-resistance exercise with sequential computerised cognitive training improved gait speed and variability in individuals with mild cognitive impairment (MCI).
- This intervention also significantly reduced falls risk at 12 months.
- Adding cognitive training to exercise yielded greater falls prevention benefits at 12 months than exercise alone.
- Vitamin D supplementation did not improve mobility and increased gait variability, suggesting potential harm.

## Introduction

Mild cognitive impairment (MCI) is a transitional stage between normal cognitive ageing and dementia, where cognitive impairment is present without a significant impact on independent living [1]. Robust evidence shows that older adults with MCI are not only more likely to progress to dementia but also exhibit mobility impairment, including slower gait [2], increased stride-to-stride fluctuations (i.e. increased variability) [3], difficulties walking while talking (i.e. dual-tasking) [3], and up to an 80% increased risk of falls compared with older individuals with normal cognition [4]. Due to the increased risk of fractures, hospitalizations and mortality [5, 6] associated with mobility problems in MCI, there is an urgent need to identify effective therapies with potential for large-scale implementation to delay both cognitive and mobility declines in older adults with MCI.

Physical exercise is a promising strategy for managing both cognitive and mobility declines in ageing [7–11]. Exercise interventions, specifically multimodal exercise (i.e. aerobic and resistance training) [12, 13] combined with cognitive training, seem to be more effective than exercise alone in enhancing cognition in older adults in general [14–16] and in those with MCI [16–20]. However, it is unknown whether these interventions, individually or in combination, can have a clinically meaningful effect on improving gait performance and reducing falls in older adults with MCI.

Another important question is whether Vitamin D, a supplement widely prescribed to older adults to maintain bone density and musculoskeletal function, could enhance the effects of exercise on gait and falls. Although cross-sectional studies have found associations between Vitamin D supplementation and cognition [21, 22], gait [23], muscle strength [24, 25], balance [24], and reduced risk of falls [26], recent meta-analyses did not confirm these benefits and even found negative effects on mobility when higher doses of Vitamin D were used [27–29].

The SYNERGIC trial [30] was a multi-site, randomised, double-blind, fractional factorial trial that tested the effects of aerobic-resistance exercise with and without cognitive training and/or Vitamin D supplementation to improve global cognition, as the primary outcome, and gait and falls as secondary outcomes in older adults with MCI. Primary results from the SYNERGIC trial [17] demonstrated that adding cognitive training to aerobic-resistance exercise significantly improved cognition, whereas adding Vitamin D did not enhance the effect. Given the interrelationship between cognition, gait, and falls in older adults [31, 32], we postulated that cognitive training and Vitamin D supplementation would further enhance the effects of exercise on gait performance, increasing propulsion and stability, and reducing the risk of falls in MCI [33–36].

## Methods

The SYNERGIC trial protocol and methods (ClinicalTrials.gov identifier: NCT02808676) are described elsewhere [30], and additional secondary findings have been previously published. In brief, the SYNERGIC trial was a double-blind, randomised trial with a fractional factorial design aimed at evaluating the effect of 20-week multidomain interventions on cognition and mobility in older adults with MCI at 6-month and 12-month endpoints. Assessments took place at baseline, post-intervention (month 6) and follow-up (month 12). The baseline visit included the collection of venous blood samples for the measurement of serum 25 (OH) Vitamin D. Specimens were stored at −80°C as aliquots immediately after centrifugation. Serum 25 (OH) Vitamin D was measured using a non-competitive electrochemiluminescence immunoassay (Cobas e 602, Roche Ltd, Basel, Switzerland).

The trial was conducted at five Canadian academic institutions: Western University (sponsor site), University of Waterloo, Wilfrid Laurier University, University of Montreal, and University of British Columbia. Ethics approval was granted by each institution’s review boards, and all participants provided written informed consent. The trial adhered to the Consolidated Standards of Reporting Trials Extension (CONSORT Extension) reporting guidelines, as extended to nonpharmacologic interventions.

### Participants

Participants were recruited from the community from 19 September 2016 to 7 April 2020. They were eligible if they were aged 60 to 85 years and met Petersen’s criteria for MCI [1], defined as follows: [1] subjective cognitive concerns; [2] objective cognitive impairment in memory, executive function, attention and/or language; [3] preserved activities of daily living; and [4] absence of dementia. Exclusion criteria included major depression, schizophrenia, substance abuse, parkinsonism, conditions affecting gait (e.g. severe osteoarthritis, previous stroke), participation in exercise programmes, and intake of Vitamin D doses greater than 1000 IU daily, cognitive enhancers, or anticholinergic medications. Full eligibility criteria are detailed in our protocol [37].

### Randomisation and masking

Participants were randomly assigned to arms in a 1:1:1:1:1 ratio using a central computer-generated random number sequence in blocks of five: arm 1 (aerobic-resistance exercise, cognitive training, and Vitamin D; Ex+Cog+VitD); arm 2 (aerobic-resistance exercise, cognitive training, and placebo Vitamin D; Ex+Cog); arm 3 (aerobic-resistance exercise, sham cognitive training, and Vitamin D; Ex+VitD); arm 4 (aerobic-resistance exercise, sham cognitive training, and placebo Vitamin D; Ex); and arm 5 (balance and toning exercise, sham cognitive training, and placebo Vitamin D; control). A research pharmacist assigned Vitamin D or placebo capsules as kits in compliance with the randomisation lists. Arm allocation was concealed from participants, who were instructed not to discuss the intervention during training sessions. Outcome assessors were blinded to allocation and were not involved in the interventions.

### Procedures

Participants in all five study arms completed group training sessions three times per week for 20 weeks. Each session lasted 90 minutes and comprised 30 minutes of cognitive training (active or sham), followed by 60 minutes of bi-modal (aerobic-resistance) or control (balance and toning) exercises. All participants received a capsule of Vitamin D (10 000 IU dose) or matching placebo three times per week, on exercise session days, for 20 weeks. Participants performed cognitive training (Neuropeak) or sham cognitive training on a computerised tablet (Apple Inc.). Neuropeak delivered two visuomotor tasks targeting working memory and attention, either separately or concurrently. The level of difficulty increased over time, and participants received individually tailored continuous feedback on performance [38]. Sham cognitive training included alternating between two tasks (touristic search and video watching, both on the same tablet used for the cognitive training) with the same exposure time as the intervention training.

The supervised progressive exercise programme combined aerobic and resistance training, broadly based on exercise prescription guidelines for older adults [39]. The exercise programme followed a predetermined and standardised volume and progression. However, session-to-session intensity was monitored and adjusted using the Borg Rating of Perceived Exertion scale [40]. Control exercises included balance, stretching, and toning exercises that did not progress in volume or intensity. Exercise groups had approximately a trainer-to-participant ratio of 1:4. All elements of the bi-modal exercise programmes were overseen by certified clinical exercise physiologists.

### Gait and falls outcomes

Gait was assessed electronically using a 6-metre electronic walkway (Zeno® walkway system) in laboratory settings by trained research assistants. Gait data recorded by the electronic walkway were processed and quantified using the ProtoKinetics Movement Analysis Software (PKMAS). The walking protocol for assessments has been previously validated and extensively tested in individuals with MCI [41]. Participants were instructed to start walking 1 metre before the start of the 6-metre electronic walkway and to stop only after crossing a marker placed on the floor 1 metre beyond the end of the walkway. Only the central 6 metres of steady walking on the walkway were recorded to avoid periods of gait acceleration and deceleration. Participants performed three trials at their preferred self-selected walking speed, one trial at fast walking speed, and three dual-task walk trials. Fast walking entailed walking as fast and safely as possible. For the dual-task conditions, participants were instructed to walk at their preferred speed without stopping while counting backwards by ones from one hundred (1 trial), naming animals (1 trial), and subtracting sevens from one hundred (1 trial). The dual-task gait cost (DTC) was used to determine the impact of a mental task on gait performance in each dual-task condition, using the DTC formula [42]. The change in gait performance from single- to dual-task estimates the effect of cognitive load on the individual’s gait performance. Additionally, changes in the number of subtractions, animals named, and subtraction errors were analysed to determine potential changes in task prioritisation strategies across trial arms.

The two main outcomes of interest for gait performance were changes in gait speed and gait variability from baseline to post-intervention (month 6). Gait speed was calculated as the distance travelled on the electronic walkway divided by its duration and is reported in cm/s. Variability was assessed using the coefficient of variation [CoV = standard deviation (SD)/mean] for both the gait cycle (i.e. stride time variability) and stride length (i.e. stride length variability) [43] within each trial. Gait speed and variability were chosen due to their strong associations with cognitive and motor decline and risk of falls [6, 44, 45].

The two main outcomes of interest for falls were incidental falls and incidental injurious falls. A fall was defined as ‘*an unexpected event in which an individual comes to rest on the ground, floor, or lower level unintentionally and not caused by stroke or seizure*’ [45, 46]. An injurious fall was defined as ‘*falls resulting in fracture, joint dislocation, laceration, bruises, abrasions, sprains, and other minor soft tissue injury*’ [46]. At baseline, participants self-reported dates and consequences of fall occurrences that happened in the past 12 months. After baseline assessment, falls were ascertained daily through a falls calendar with fields for a detailed explanation of the event. Participants were instructed to fill in details related to fall events, including the reason for the fall (e.g. trip, loss of balance, stumble, misstep, syncope, etc.), injuries sustained, type of injuries, and need for medical care and/or hospitalisation during the 12-month study period. Falls were then tallied by a trained assessor at the 6-month and 12-month assessments.

### Statistical analysis

**Figure 1:**
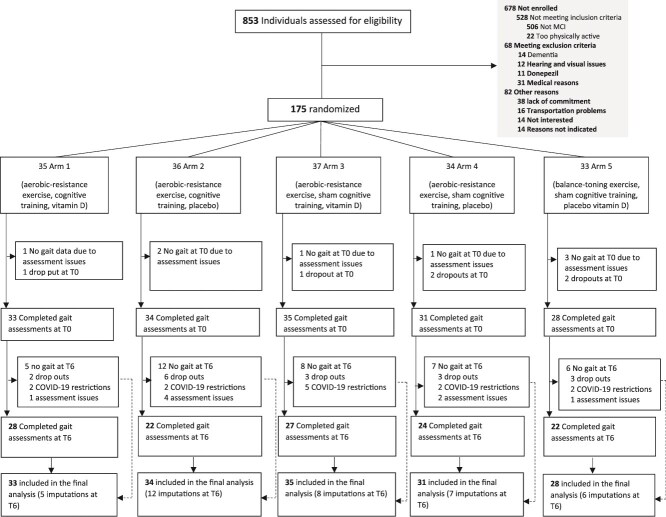
SYNERGIC Trial Consortium flowchart and sample eligible for gait and falls analyses (secondary outcomes).

To adhere to the intention-to-treat principle, all individuals with available gait data at baseline (pre-intervention) were analysed according to randomisation (Figure 1). One-way analysis of variance (ANOVA) was used to compare 6-month changes in gait outcomes across the intervention arms. A Tukey post-hoc test was performed to compare differences between the intervention arms. To assess the potential synergistic effects of adding cognitive training and Vitamin D to aerobic-resistance exercise, we conducted ‘at the margin’ analyses by pooling arms in pre-specified combinations, as outlined in the pre-specified analysis plan [17]. For ‘at the margin’ analyses, gait outcomes were analysed using a linear mixed model with repeated measures, including intervention arm, time and time-by-intervention-arm interaction. To facilitate the interpretation of intervention effects, effect sizes (d) were calculated as the differences divided by the SD.

 The number of falls was analysed using a Quasi-Poisson regression, controlling for overdispersion (φ = 1.6) [11] and baseline falls, following ‘at the margin’ analyses for the pre-specified comparison to assess potential synergistic effects of adding cognitive training and Vitamin D to exercise on falls rates. Results are presented as incidence rate ratios (IRR).

The proportion of individuals with missing gait data at month 6 did not significantly differ across arms (*P* > .40), suggesting that data missingness happened at random. For this reason, and in accordance with the intention to treat principle, mean gait performance recorded at month 6 was imputed only for participants with complete gait data at baseline, for computational ease (Figure 1). Due to a few missing data on falls (<10%) across visits, the last observation (i.e. falls reported at baseline) carried forward method was used to prevent misleading biases towards zero falls at month 6. Also, there were no statistically significant differences (*P* > .30) across the trial’s arms for age, sex, education, global cognition, mood, functionality, and the total number of medications taken (Table 1). Due to a significant amount of missing gait data, intervention effects at the follow-up (month 12) were assessed for the falls only. The missing gait data arose from COVID-19 restrictions, which prevented in-person gait assessments during the follow-up.

**Table 1:** Participants characteristics in each arm at baseline (T0).

	Total	Arm 1 (*N* = 33)	Arm 2 (*N* = 34)	Arm 3 (*N* = 35)	Arm 4 (*N* = 31)	Arm 5 (*N* = 28)
	(*N* = 161)	Ex + Cog + Vit D mean (SD) or N (%)	Ex + Cog mean (SD) or N (%)	Ex + Vit D mean (SD) or N (%)	Ex Mean (SD) or N (%)	Control mean (SD) or N (%)
Age in years	73.08 (6.59)	73.49 (6.02)	72.27 (7.19)	73.71 (7.82)	72.36 (5.25)	73.61 (6.45)
Sex, women, n (%)	83 (51.55)	20 (60.6%)	15 (44.11%)	18 (51.4%)	14 (45.16%)	16 (57.14%)
Education in years	15.27 (3.52)	14.52 (2.55)	15.38 (3.31)	14.19 (3.26)	15.37 (3.15)	17.29 (4.67)
PASE, (0–700)	112.42 (57.70)	123.76 (56.17)	108.71 (60.62)	118.75 (64.73)	101.10 (51.16)	108.41 (54.41)
MoCA, (0–30)	22.57 (3.27)	23.12 (3.40)	22.47 (3.74)	22.43 (3.35)	22.45 (3.21)	22.36 (2.61)
IADL(0–23)	22.2 (1.73)	22.19 (1.68)	22.36 (1.54)	22.09 (2.09)	22.07 (1.65)	22.37 (1.69)
Number of comorbidities	4.36 (2.41)	4.59 (2.66)	4.35 (2.14)	4.20 (2.76)	4.26 (2.40)	4.40 (2.08)
Medications taken	7.1 (3.71)	3.81 (3.82)	4.37 (4.80)	3.18 (3.72)	3.50 (4.11)	2.96 (4.22)
ADAS-Cog13	14.14 (5.83)	14.01 (7.00)	13.58 (6.08)	15.73 (6.27)	14.34 (5.16)	12.78 (3.74)
Falls baseline, n (%)	49 (30.4)	10 (29.4%)	5 (14.7%)	10 (27%)	11 (35%)^†^	13 (46%)^†^
Injurious falls baseline, n (%)	21 (13.04)	4 (11.7%)	3 (8.8%)	5 (13.8%)	6 (20%)	3 (10.7%)
Recurrent fallers, n(%)	8 (4.96)	3 (9.09)	0 (0)	2 (5.71)	1 (3.22)	2 (7.14)
Gait speed, cm/s	117.15 (21.75)	118.77 (24.68)	122.03 (20.26)	112.56 (18.96)	116.49 (23.97)	115.84 (20.65)
Fast gait speed, cm/s	157.85 (28.18)	156.35 (28.00)	164.32 (30.14)	153.97 (25.15)	159.34 (32.90)	154.99 (24.16)

Note: MoCA, Montreal cognitive assessment; IADL, Instrumental activities of daily living Lawton & Brody scale; ADAS-Cog13, Alzheimer’s disease assessment scale 13 items; PASE, Physical Activity Scale for the Elderly. ^†^One missing value at baseline for participants with gait data; two missing values at baseline.

To assess whether physical activity levels were affected by COVID-19 restrictions during the follow-up (month 12), the Friedman rank sum test was used to compare Physical Activity Scale for the Elderly (PASE) scores across baseline, post-intervention, and follow-up.

Interpretation of statistical tests was based on a two-sided 5% significance level. All analyses were conducted in SPSS version 29.0 (SPSS Inc.) and JASP Team (2024) version 0.18.3 (computer software). Sample size was calculated to detect a moderate effect size of 0.5 based on the primary cognitive outcome, ADAS-Cog-13, and was deemed to be sufficient for secondary mobility outcomes if the combined intervention could reach a moderate-to-large effect size of ~0.5.

## Results

### Participant characteristics

Of the 853 individuals screened for eligibility, 175 (20%) were randomised [mean (SD) age, 73.1 (6.6) years; 83 (51.6%) female; and predominantly White (83.2%); Figure 1].

Table 1 summarises participants’ baseline demographic, clinical, cognitive, and physical characteristics. From the 175 participants randomised, a total of 161 had gait data recorded for the purposes of this analysis. After baseline assessments, 17 participants withdrew from the study. Twenty-four participants could not complete their gait assessment at month 6 for several reasons, the most common being COVID-19 related lockdowns and assessment errors (see Figure 1 for details). Although the number of participants who could not complete the gait assessment at month 6 differed by site (one from University of British Columbia, three from Wilfrid Laurier University, seven from University of Montreal, and two from University of Waterloo), the number of individuals with missing data for gait at month 6 did not significantly differ across arms (*P* > .40). Additional characteristics of gait measures in the sample are summarised in Appendix 1. The follow-up (month 12) assessment was done virtually for 37 individuals due to COVID-19 restrictions. Therefore, gait assessments were not performed on these 37 participants at 12 months.

### Effects on gait performance

**Figure 2:**
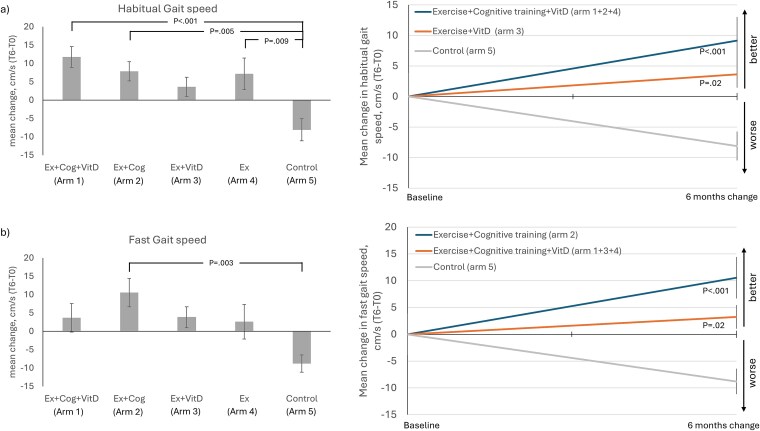
Intervention effects for gait speed, habitual (a) and fast conditions (b). Bar plots show the gait speed mean change across arms with post-hoc results comparing each arm with control. Right panels (line graphs) show comparisons between pooled arms with control (arm 5) showing their respective slopes of change and standard errors (SEs).

Figure 2 shows post-intervention changes with their respective effect sizes for all gait variables. Gait speed (F_4,156_ = 5.50, *P* < .001) and fast gait speed (F_4,156_ = 3.39, *P* = .01) increased in all intervention arms, while the control arm showed a gait speed decline (Appendix 2). Table 2 presents the post-hoc pairwise comparison across arms. A significant increase in gait speed was seen in arm 1 [Ex+Cog+VitD; mean difference = 19.87 cm/s; 95% confidence interval (CI): 7.4, 32.34; *P* < .001; d = 1.13] arm 2 (Ex+Cog; mean difference = 15.94 cm/s; 95% CI: 3.56, 28.32; *P* = .005; d = 0.90) and arm 4 (Ex; mean difference = 15.28 cm/s; 95% CI: 2.63, 27.93; *P* = .009; d = 0.87) compared with control (arm 5). For fast gait speed, arm 2 (Ex+Cog) showed a significant increase (mean difference = 19.33 cm/s; 95% CI: 4.7, 33.97; *P* = .003, d = 0.93) compared to control. Table 3 presents the linear mixed model analyses using ‘at the margin’ approach, which revealed that pooled arms 1 + 3 + 4 (mean difference = 12.02 cm/s; 95% CI: 1.53, 22.5; *P* = .02; d = 0.58; Figure 2) improved fast gait speed relative to control.

**Table 2:** Post-hoc pairwise comparison of mean change in gait speed outcomes between groups.

	Arm 2 (Ex+Cog)			Arm 3 (Ex+VitD)			Arm 4 (Ex)			Arm 5 (control)		
	Mean difference (95% CI)	*P*-value	d	Mean difference (95% CI)	*P*-value	d	Mean difference (95% CI)	*P*-value	d	Mean difference (95% CI)	*P*-value	d
Gait speed												
Arm 1 (Ex+Cog+VitD)	3.92 (−7.92, 15.78)	.89	0.22	8.13 (−3.63, 19.91)	.32	0.46	4.58 (−7.55, 16.72)	.84	0.26	19.87 (7.4, 32.34)	**<.001**	1.13
Arm 2 (Ex+Cog)				4.2 (−7.47, 15.89)	.86	0.24	0.65 (−11.39, 12.7)	.99	0.04	15.94 (3.56, 28.32)	**.005**	0.9
Arm 3 (Ex+VitD)							−3.55 (−15.51, 8.41)	.93	−0.20	11.73 (−0.56, 24.04)	.07	0.66
Arm 4 (Ex)										15.28 (2.63, 27.93)	**.009**	0.87
Fast gait speed												
Arm 1 (Ex+Cog+VitD)	−6.86(−20.87, 7.15)	.66	−0.33	−0.13 (−14.04, 13.78)	.99	−0.01	1.07 (−13.26, 15.42)	.99	0.05	12.47 (−2.26, 27.21)	.14	0.6
Arm 2 (Ex+Cog)				6.73 (−7.07, 20.54)	.66	0.32	7.93 (−6.3, 22.18)	.54	0.38	19.33 (4.7, 33.97)	**.003**	0.93
Arm 3 (Ex+VitD)							1.20 (−12.93, 15.35)	.99	0.06	12.6 (−1.93, 27.14)	0.12	0.6
Arm 4 (Ex)										11.4 (−3.55, 26.35)	0.22	0.54

Note: In bold, all statistically significant effects at *P* < .05.

**Table 3:** Effect of exercise (aerobic-resistance training) with and without cognitive training and vitamin D on gait speed at 6-month.

	All Interventions vs control arms 1 + 2 + 3 + 4 vs. arm 5(ref)				Adding cognitive intervention to exercises arms 1 + 2 vs. arm 3 + 4(ref)				Adding vitamin D intervention arms 1 + 3 vs. arm 2 + 4(ref)				Multidomain intervention arm 1 vs. arm 5(ref)			
	Mean change (SE) within group	Mean difference between groups (95% CI)	*P*-value	d	Mean change (SE) within group	Mean difference between groups (95% CI)	*P*-value	d	Mean change (SE) within group	Mean difference between groups (95% CI)	*P*-value	d	Mean change (SE) within group	Mean difference between groups (95% CI)	*P*-value	d
Gait speed		15.66 (8.42, 22.90)	**<.001**	−0.89		4.48 (−1.66, 10.61)	.09	−0.25		0.05 (−6.12, 6.24)	.96	−0.003		19.87 (11.61, 28.14)	**< 0.001**	−1.24
Comparison group	7.55 (1.56)				9.77 (1.92)				7.58 (1.97)				11.77 (2.82)			
Reference group	−8.11 (3.01)				5.30 (2.44)				7.53 (2.45)				−8.11 (3.01)			
Fast gait speed		14.01 (5.48, 22.55)	**<.001**	−0.67		3.92 (−3.66, 11.50)	.20	−0.18		3.01 (−10.61, 4.59)	.40	0.14		12.48 (2.97, 21.98)	**0.003**	−0.68
Comparison group	5.22 (1.92)				7.17 (2.76)				3.75 (2.38)				3.69 (3.89)			
Reference group	−8.79 (2.36)				3.25 (2.66)				6.76 (3.04)				−8.79 (2.36)			

Note: In bold, all statistically significant effects at *P* < .05. d, effect size.

Marginal means and SEs obtained from linear mixed models are reported for within-group differences. Between-group differences were assessed using the interaction between time x intervention arm. Lower scores indicate cognitive improvement.

**Figure 3:**
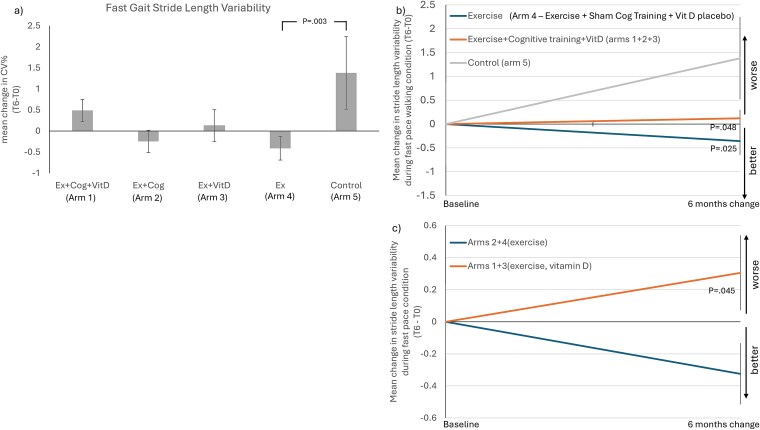
(a) Bar plots show mean change in Gait variability—stride length—across arms; (b) Comparison between pooled arms 1 + 2 + 3 (mixed interventions), arm 4 (exercises) and arm 5 (control) based on post-hoc results and their respective slopes of change and SEs. (c) Effect of adding Vitamin D to exercises on stride length variability compared with exercises with placebo vitamin D and their respective slopes of change and SEs.

Stride length variability in fast gait, a measure of gait consistency, decreased significantly in arm 4 (Ex) compared with control (mean difference = −1.79%; 95% CI: −3.57, -0.004; *P* = .04; d = −0.72; Appendix 3 and Figure 3). Adding Vitamin D to aerobic-resistance exercise (arms 1 + 3) showed an increase in stride length variability in fast gait compared with exercise arms without Vitamin D (arms 2 + 4; mean difference = −0.63%; 95% CI: −1.22, −0.03; *P* = .045; d = −0.36; Appendix 4 and Figure 3).

There were no significant differences between arms for DTC and the dual-task cognitive tasks, including the total numbers counted, total serial 7s subtractions, and animals named or errors (Appendices 5 and 6).

### Effects on falls and injurious falls

Appendix 7 shows the distribution of falls and injurious falls across the five intervention arms at 6-month and 12-month endpoints.

#### Effect at 6-month endpoint

Poisson regression models revealed that the arms receiving active interventions (pooled arms 1 + 2 + 3 + 4) experienced a 35% and 62% non-statistically significant reduction in the rate of falls (IRR = 0.65; 95% CI: 0.32, 1.42; *P* = .25) and injurious falls (IRR = 0.38; 95% CI: 0.15, 1.05; *P* = .05), respectively, compared with control (Table 4). Arms receiving aerobic-resistance exercise and Vitamin D (arms 1 + 3) experienced a 31% increase in injurious falls compared with the arms receiving aerobic-resistance exercise without Vitamin D (arms 2 + 4; IRR = 1.31; 95% CI: 0.41, 4.37; *P* = .64; Table 4). Appendix 8 shows the effect of individual active arms on falls outcomes. Overall, a non-statistically significant reduction in falls and injurious falls was found across all the arms, with arm 2 interventions (Ex+Cog) showing the largest effect on reducing falls by 66% (IRR = 0.34; 95% CI: 0.08, 1.10; *P* = .09) and injurious falls by 87% (IRR = 0.13; 95% CI: 0.008, 0.74; *P* = .06).

**Table 4:** Effect of pooled arms (aerobic-resistance exercise intervention with and without cognitive training and vitamin D) on falls outcomes at 6-month.

	Falls		Injurious falls	
	IRR (95% CI)	*P*	IRR (95% CI)	*P*
Exercise Intervention				
Arm 1 + 2 + 3 + 4 (exercise)	0.65 (0.32, 1.42)	.25	0.38 (0.15, 1.05)	.05
Arm 5 (control)	Reference		Reference	
Adding cognitive intervention				
Arm 1 + 2 (exercise, cognitive training)	0.64 (0.28, 1.40)	.28	0.36 (0.08, 1.20)	.12
Arm 3 + 4 (exercise)	Reference		Reference	
Adding vitamin D intervention				
Arm 1 + 3(exercise, vitamin D)	0.83 (0.38, 1.77)	.64	1.31 (0.41, 4.37)	.64
Arm 2 + 4 (exercise)	Reference		Reference	
Multidomain intervention				
Arm 1 (exercise, cognitive training, vitamin D)	0.27 (0.04, 1.08)	.10	0.27 (0.04, 1.08)	.10
Arm 5 (control)	Reference		Reference	

Note: IRR, Incident rate ratio; CI, Confidence interval.

#### Effect at 12-month endpoint

Falls rate at month 12 was significantly lower in arm 1 (Ex+Cog+VitD; IRR = 0.13; 95% CI: 0.02, 0.53; *P* = .01) and arm 2 (Ex+Cog; IRR = 0.24; 95% CI: 0.05, 0.77; *P* = .02) compared to the control (Table 5). Similarly, arms receiving aerobic-resistance exercise (pooled arms 1 + 2 + 3 + 4) showed a significant reduction in falls rate at month 12 (IRR = 0.28; 95% CI: 0.13, 0.64; *P* = .002; Table 6).

**Table 5:** Effect of each intervention arm on falls outcomes at 12-month.

	Falls		Injurious falls	
	IRR (95% CI)	*P*-value	IRR (95% CI)	*P*-value
Arm 1 (Ex+Cog+VitD)	**0.13 (0.02, 0.53)**	.01	0.49 (NA)	.33
Arm 5 (control)	Reference		Reference	
Arm 2 (Ex+Cog)	**0.24 (0.05, 0.77)**	.02	0.22 (NA)	.18
Arm 5 (control)	Reference		Reference	
Arm 3 (Ex+VitD)	0.33 (0.10, 0.95)	.05	1.04 (NA)	.94
Arm 5 (control)	Reference		Reference	
Arm 4 (Ex)	0.43 (0.13, 1.17)	.11	NA	NA
Arm 5 (control)	Reference		Reference	

Note: In bold, all statistically significant effects at *P* < .05. IRR, Incident rate ratio; CI, Confidence interval.

NA, not available due to missing data.

**Table 6:** Effect of pooled arms exercise (aerobic-resistance training with and without cognitive training and vitamin D) on falls outcomes at 12-month.

	Falls		Injurious falls	
	IRR(95% CI)	*P*-value	IRR(95% CI)	*P*-value
Exercise Intervention				
Arm 1 + 2 + 3 + 4 (exercise)	**0.28 (0.13, 0.64)**	.002	0.43 (0.14, 1.58)	.16
Arm 5 (control)	Reference		Reference	
Adding cognitive intervention				
Arm 1 + 2 (exercise, cognitive training)	0.51 (0.19, 1.24)	.15	0.61 (0.13, 2.44)	.5
Arm 3 + 4 (exercise)	Reference		Reference	
Adding vitamin D intervention				
Arm 1 + 3(exercise, vitamin D)	0.71 (0.29,1.68)	.44	6.74 (1.14, 141.4)	.08
Arm 2 + 4 (exercise)	Reference		Reference	
Multidomain intervention				
Arm 1 (exercise, cognitive training, vitamin D)	**0.13 (0.01, 0.66)**	.04	0.40 (0.05, 2.01)	.29
Arm 5 (control)	Reference		Reference	

Note: In bold, all statistically significant effects at *P* < .05; IRR, Incident rate ratio; CI, Confidence interval.

### Physical activity during follow-up

Exercise and physical activity levels were not maintained at the same level during the 6-month follow-up; however, the level of physical activity in those that were followed during COVID-19 restrictions was not significantly different from their level before the restrictions, except for arm 1 (Appendix 9).

## Discussion

The SYNERGIC trial is, to the best of our knowledge, the first to investigate the effect of adding cognitive training and Vitamin D to aerobic-resistance exercises on gait performance and falls rate in older adults with MCI. The combination of physical exercise with cognitive training yielded a greater effect on improving gait speed, fast gait speed, and reducing gait variability—a marker of gait consistency—compared with the single interventions, suggesting a synergistic effect.

Although not statistically significant, adding cognitive training to aerobic-resistance exercise resulted in an 83% falls rate reduction at 6 months, an effect that was maintained and reached significance at the 12-month endpoint. Vitamin D did not enhance these effects and, interestingly, combining Vitamin D with exercise resulted in less improvement in gait speed and increased gait instability compared to exercise alone. Overall, our results suggest that adding cognitive training to aerobic-resistance exercise, but not Vitamin D, results in clinically meaningful improvement in gait performance—speed and gait consistency—and in a reduction of falls in older adults with MCI.

The addition of cognitive training to physical exercise showed a larger effect in improving gait speed and fast gait speed. A possible explanation for this specific effect may be related to an enhanced cortical control of gait by combining exercise with cognitive training [47]. This increased cortical control may be necessary for controlling propulsion demands [36] and compensating for age-related sensorimotor deficits [48, 49]. Prefrontal brain regions are strongly associated with higher-level cognition for behavioural regulation, and therefore, these regions may benefit most from cognitive training focused on attention and executive function. This assumption is also supported by a statistically significant reduction in dual-tasking effects in the aerobic-resistance exercise arm exposed to cognitive training, compared to the arms receiving aerobic-resistance exercise without cognitive training in our trial. These interpretations, however, should be considered cautiously because the interventions did not significantly improve dual-task gait relative to the control arm. Mechanistically, since the SYNERGIC trial showed improved semantic and episodic memory performance in those receiving exercises with cognitive training, it is also important to consider the potential role of the hippocampus in gait speed improvement. Aligned with this hypothesis, we found that older individuals with MCI who were at higher risk of falls and had worst gait performance during obstacle negotiations had lower hippocampal volumes on brain MRI [50]. These results together reinforce the notion that exercise combined with cognitive training may help improve voluntary control of gait performance.

Adding Vitamin D to aerobic-resistance exercise diminished the benefits of exercise and led to less improvement in gait speed compared with aerobic-resistance exercise alone. This aligns with a recent systematic review and meta-analysis that indicated that Vitamin D may have a detrimental effect on muscle strength and may increase risk of falls [28, 29]. Similarly, it has been suggested that a high dose of Vitamin D may negatively affect cognitive processing and speed, which might explain this result in our study [30, 51]. To note, our Vitamin D supplementation reached an equivalent dose of 4000 IU/day, which is considered the safe upper limit and not a ‘high’ supplemental dose.

In agreement with previous studies [7, 11, 52], all intervention arms that included aerobic-resistance exercise were associated with a reduced rate of injurious falls. When comparing the IRR between intervention arms and control group, aerobic-resistance exercise combined with cognitive training showed a larger effect. A potential explanation for this specific protective effect, for injuries when a fall occurred, is that exercise coupled with cognitive training may not only improve muscle strength and balance but also enhance cognitive processing speed, which in turn can enhance postural reflexes and defence reflexes mechanisms [53], which are triggered to prevent falls and de-accelerate ground contact [54].

In addition, Neuropeak—the cognitive training programme used in the SYNERGIC trial—mainly focused on executive functions and working memory [38], which may have also improved navigation control [55, 56] and reduced encounters with postural hazards, trips, and stumbles [57] as well as other situations that could trigger a fall [58]. In support of these assumptions, an experimental study [59] revealed that higher-level cognition helped coordinate the sequence and timing of the postural reactions, ultimately making them more effective in preventing and containing balance instabilities. Overall, our results suggest that adding cognitive training sequentially to aerobic-resistance exercise may effectively reduce the rate of injurious falls in older adults with MCI.

### Limitations

Important limitations need to be acknowledged. Our sample was not powered for the falls analysis, since the sample size of the SYNERGIC trial was estimated based on a meaningful change in the primary cognitive outcome, the ADAS-Cog 13. Therefore, despite the large effect size observed, particularly for the falls analysis, confidence intervals were quite wide, thus changes in falls at the 6-month endpoint were not statistically significant. Our sample predominantly consisted of White individuals with post-secondary education, which limits the generalizability of our findings [60]. Only 5% of our participants were Vitamin D deficient, which may have limited the ability to detect any positive effect of Vitamin D supplementation. Emerging evidence shows that providing Vitamin D to those who are not deficient is not helpful for mobility and falls outcomes [61]. Finally, due to the nature of these secondary outcomes analyses, multiplicity was not adjusted for in the linear mixed model; thus, our findings should be interpreted with caution given the multiple comparisons and chance of type I error.

## Conclusion

A combination of aerobic-resistance exercise significantly improves gait speed and gait variability at 6 months, and reduces falls at 12 months in older adults with MCI. The addition of sequential cognitive training yielded a larger effect in improving gait speed and reducing injurious falls. Adding Vitamin D to aerobic-resistance exercise produced mixed and negative effects on gait outcomes, not supporting their use for improving mobility and preventing falls in older adults with MCI. As exercise paired with cognitive training were feasible to conduct in older adults with cognitive impairment and with minimal equipment, they have the potential to increase the treatment opportunities available to address the growing problem of falls and fall-related injuries in older adults with MCI.

## Supplementary Material

Supplementary_materials_afaf242

## Data Availability

Data may be available upon request to the corresponding author.
