# Supplementary material for: Synergistic effects of exercise, cognitive training and vitamin D on gait performance and falls in mild cognitive impairment—secondary outcomes from the SYNERGIC trial
Source: Age Ageing. 2025 Sep 12;54(9):afaf242. doi: 10.1093/ageing/afaf242 (PMC12445844; doi:10.1093/ageing/afaf242)
Supplement: Supplementary_materials_afaf242 [file supplementary_materials_afaf242.docx]

**Synergistic Effects of Exercise, Cognitive Training, and Vitamin D on Gait Performance and Falls in Mild Cognitive Impairment - Secondary Outcomes from the SYNERGIC Trial**

**Appendices**

**Table of Contents**

Appendix 1 – Gait variables characteristics at baseline and post-intervention (month 6)

Appendix 2. Means change in gait outcomes at post-intervention (month 6) across arms

Appendix 3. Post-hoc pairwise comparison of mean change in gait outcomes between arms

Appendix 4. Effect of exercise (aerobic-resistance training) intervention with addition of cognitive training and vitamin D at 6-month on Dual-Task Cost (DTC)

Appendix 5 – One-way ANOVAs comparing the mean change of the cognitive task results during the dual task conditions

Appendix 6 – Effect of exercise (aerobic-resistance training) intervention with addition of cognitive training and vitamin D at 6-month end point in the cognitive tasks

Appendix Table 7 – Falls distribution across arms at baseline (T0), month 6 (T6), and month 12 (T12)

Appendix 8 – Effect of aerobic-resistance exercise intervention arms on falls rate at 6-month

Appendix 9 – The mean Physical Activity Scale for the Elderly (PASE) score at baseline, 6-month, and 12-month endpoints across arms

Appendix 1 – Gait variables characteristics at baseline and post-intervention (month 6)

| Variables | Time points | Arm 1  (Ex+Cog+VitD)  Mean(SD) | Arm 2  (Ex+Cog)  Mean(SD) | Arm 3  (Ex+VitD)  Mean(SD) | Arm 4  (Ex)  Mean(SD) | Arm 5  (Control)  Mean(SD) |
| --- | --- | --- | --- | --- | --- | --- |
| Gait speed, cm/s | T0 | 118.77 (24.68) | 122.03 (20.26) | 112.56 (18.96) | 116.49 (23.97) | 115.84 (20.65) |
|  | T6 | 130.54 (19.65) | 129.86 (17.72) | 116.19 (15.89) | 123.67 (17.65) | 107.73 (19.18) |
| Fast gait, cm/s | T0 | 156.35 (28.00) | 164.32 (30.14) | 153.97 (25.15) | 159.34 (32.90) | 154.99 (24.16) |
|  | T6 | 160.04 (27.18) | 174.87 (20.74) | 157.79 (19.19) | 161.95 (28.88) | 146.20 (25.22) |
| Gait speed counting backwards by 1s, cm/s | T0 | 110.64 (23.52) | 106.72 (20.31) | 105.57 (25.55) | 109.67 (25.20) | 99.12 (34.05) |
|  | T6 | 122.97 (22.14) | 124.40 (19.07) | 110.22 (17.18) | 110.05 (25.61) | 103.71 (22.07) |
| Gait speed serial 7s, cm/s | T0 | 99.12 (34.05) | 91.53 (30.27) | 94.28 (21.18) | 91.88 (26.48) | 94.04 (25.40) |
|  | T6 | 104.12 (28.15) | 105.85 (23.51) | 95.94 (19.11) | 103.98 (24.53) | 92.64 (22.07) |
| Gait speed naming animals, cm/s | T0 | 102.62 (29.99) | 103.48 (26.15) | 97.12 (22.99) | 97.82 (28.20) | 99.42 (24.74) |
|  | T6 | 3.09 (1.70) | 3.51 (2.74) | 3.54 (2.10) | 4.68 (5.91) | 3.39 (1.49) |
| DTC counting backward, % | T0 | 8.04 (13.01) | 8.99 (13.84) | 4.87 (12.27) | 8.80 (15.53) | 5.99 (8.00) |
|  | T6 | 5.96(7.73) | 4.12 (6.88) | 5.20 (6.43) | 11.45 (13.67) | 3.82 (11.79) |
| DTC serial 7s, % | T0 | 17.54 (18.91) | 24.63 (23.65) | 15.53 (16.90) | 20.18 (19.56) | 18.90 (15.07) |
|  | T6 | 20.56 (17.02) | 18.49 (15.42) | 17.24 (11.93) | 14.90 (18.70) | 13.68 (16.64) |
| DTC naming animals, % | T0 | 14.26 (13.83) | 14.83 (17.74) | 13.31 (16.40) | 15.74 (18.32) | 14.77 (9.76) |
|  | T6 | 11.88 (10.53) | 10.68 (10.51) | 13.60 (10.44) | 9.44 (18.18) | 9.40 (11.24) |
| Gait stride time variability, % | T0 | 3.34 (2.72) | 2.48 (1.39) | 2.92 (1.68) | 2.36 (0.82) | 2.91 (1.77) |
|  | T6 | 3.10 (3.73) | 3.22 (1.56) | 2.89(1.46) | 2.62 (0.73) | 3.48 (2.54) |
| Fast gait stride time variability, % | T0 | 2.45 (1.93) | 2.71 (3.17) | 2.95 (3.25) | 2.56 (1.36) | 3.35 (3.20) |
|  | T6 | 4.03 (5.07) | 2.15 (1.25) | 2.79 (2.46) | 3.22 (2.48) | 4.62 (8.09) |
| Counting backward by 1s stride time variability, % | T0 | 4.73 (7.60) | 2.89 (2.39) | 2.81 (1.26) | 6.26 (14.34) | 2.99 (1.58) |
|  | T6 | 2.53 (0.98) | 6.52 (10.46) | 4.01 (4.45) | 4.38 (4.39) | 10.72 (30.90) |
| Serial 7s subtractions stride time variability, % | T0 | 4.87 (4.23) | 2.40 (1.46) | 2.53 (0.98) | 3.27 (2.84) | 6.07 (10.25) |
|  | T6 | 10.99 (26.25) | 3.74 (2.36) | 3.87 (3.32) | 5.42 (7.85) | 3.92 (2.09) |
| Naming animals stride time variability, % | T0 | 3.90(3.15) | 7.73 (9.46) | 4.05 (5.07) | 5.06 (4.39) | 4.63 (4.59) |
|  | T6 | 3.09 (1.70) | 3.62 (2.76) | 3.56 (2.10) | 4.57 (5.93) | 3.29 (1.50) |
| DTC counting backward stride time variability, % | T0 | 98.68 (340.03) | 26.64 (72.87) | 20.77 (72.82) | 174.55 (627.61) | 24.38 (71.88) |
|  | T6 | 29.77 (76.04) | -23.08 (33.90) | 4.33 (63.27) | 26.38 (91.48) | 82.09 (243.88) |
| DTC serial 7s stride time variability, % | T0 | 84.86 (144.95) | 235.97 (359.09) | 52.64 (137.22) | 124.91 (186.80) | 99.89 (270.10) |
|  | T6 | 429.24 (1305.88) | 85.43 (159.14) | 59.19 (158.78) | 65.68 (136.12) | 368.52 (1612.54) |
| DTC naming animals stride time variability, % | T0 | 55.13 (151.54) | 75.94 (119.15) | 56.57 (120.61) | 147.22 (446.08) | 56.92 (91.10) |
|  | T6 | 38.67 (95.04) | 15.86 (55.68) | 65.83 (184.34) | 73.14 (176.70) | 21.30 (68.60) |
| Gait stride length variability, % | T0 | 4.23 (7.89) | 2.99 (1.26) | 3.47 (2.51) | 2.91(1.13) | 3.25 (1.98) |
|  | T6 | 2.94 (1.26) | 3.17 (1.15) | 3.33 (1.18) | 3.21 (0.89) | 3.84 (2.53) |
| Fast gait stride length variability, % | T0 | 2.57 (1.13) | 2.68 (1.62) | 2.90 (2.03) | 2.85 (1.42) | 3.29 (2.53) |
|  | T6 | 3.06 (1.37) | 2.43 (1.13) | 3.03 (1.52) | 2.44 (0.69) | 4.67 (4.54) |
| Counting backward by 1s stride length variability, % | T0 | 3.28 (1.77) | 3.66 (2.91) | 3.26 (1.91) | 3.65 (1.89) | 3.61 (3.04) |
|  | T6 | 7.58 (20.82) | 3.71 (1.83) | 5.79 (8.62) | 4.17 (2.16) | 4.57 (2.40) |
| Serial 7s subtractions stride length variability, % | T0 | 3.88 (1.99) | 2.84 (1.22) | 7.58 (20.82) | 3.54 (2.04) | 3.63 (1.50) |
|  | T6 | 4.61 (3.56) | 4.01 (2.35) | 5.15 (4.12) | 4.67 (2.94) | 3.81 (1.76) |
| Naming animals stride length variability, % | T0 | 4.50 (2.56) | 6.49 (9.42) | 4.69 (5.63) | 4.08 (2.25) | 3.93 (2.47) |
|  | T6 | 3.26 (1.19) | 4.15 (2.97) | 4.11 (1.628) | 4.04 (1.65) | 3.96 (1.43) |
| DTC counting backward stride length variability, % | T0 | 30.94 (100.13) | 35.01 (90.44) | 10.30 (52.27) | 43.47 (90.30) | 21.61 (95.32) |
|  | T6 | 46.05 (87.37) | -7.83 (39.20) | 114.61 (511.88) | 14.01 (65.88) | 16.39 (78.37) |

Note: DTC, Dual task gait cost

Appendix 2. Means change in gait outcomes at post-intervention (month 6) across arms

|  | Arm 1 (Ex+Cog+VitD)  mean (SE) | Arm 2 (Ex+Cog)  mean (SE) | Arm 3 (Ex+VitD)  mean (SE) | Arm 4  (Ex)  mean (SE) | Arm 5  (Control)  mean (SE) | F | p |
| --- | --- | --- | --- | --- | --- | --- | --- |
| Gait speed | 11.76 (2.81) | 7.83 (2.61) | 3.63 (2.6) | 7.18 (4.3) | -8.1 (3.01) | **5.50** | **<.001** |
| Fast gait speed | 3.68 (3.89) | 10.54 (3.88) | 3.81 (2.86) | 2.60 (4.7) | -8.79 (2.35) | **3.39** | **0.01** |
| DTC gait speed counting backwards by 1s | -2.08 (2.01) | -4.86 (1.99) | 0.32 (1.73) | 2.64 (1.74) | -2.17 (2.31) | 2.13 | 0.07 |
| DTC gait speed naming animals | 3.01 (3.51) | -6.14 (2.66) | 1.70 (2.27) | -5.28 (3.46) | -5.22 (4.09) | 1.91 | 0.11 |
| DTC gait speed serial 7s | -2.38 (2.28) | -4.14 (2.53) | 0.29 (2.09) | -6.30 (3.28) | -5.36 (2.49) | 1.07 | 0.37 |
| Gait stride time variability | -0.24 (0.61) | 0.74 (0.31) | -0.03 (0.38) | 0.26 (0.16) | 0.56 (0.56) | 0.91 | 0.45 |
| Stride time variability in fast gait | 1.57 (0.98) | -0.56 (0.61) | -0.16 (0.73) | 0.66 (0.5) | 1.26 (1.72) | 0.93 | 0.44 |
| DTC stride time variability counting backwards by 1s | -68.91 (61.21) | -49.72 (14.07) | -16.44 (16.52) | -148.16 (110.08) | 57.71 (46.98) | 1.50 | 0.20 |
| DTC stride time variability serial 7s | 344.38 (221.09) | -150.53 (48.41) | 6.54 (38.6) | -59.22 (33.08) | 268.63 (309.59) | 0.77 | 0.54 |
| DTC stride time variability naming animals | -16.45 (31.06) | -60.08 (18.29) | 9.26 (25.59) | -74.07 (72.99) | -35.62 (21.05) | 1.77 | 0.13 |
| Gait stride length variability | -1.29 (1.43) | 0.18 (0.22) | -0.14 (0.47) | 0.29 (0.23) | 0.59 (0.56) | 0.95 | 0.43 |
| Stride length variability in fast gait | 0.49 (0.26) | -0.24 (0.26) | 0.12 (0.38) | -0.4 (0.28) | 1.38 (0.86) | **2.43** | **0.04** |
| DTC stride length variability counting backwards by 1s | 15.11 (19.72) | -42.83 (17.05) | 104.3 (86.43) | -29.45 (18.88) | -5.22 (24.56) | 1.72 | 0.14 |
| DTC stride length variability serial 7s | 38.36 (28.33) | -129.06 (67.02) | 37.8 (61.41) | -26.10 (25.08) | 28.07 (27.32) | 2.32 | 0.056 |
| DTC stride length variability naming animals | -33.17 (21.72) | -16.49 (26.22) | -48 (22.05) | -43.92 (20.15) | -12.40 (24.35) | 0.47 | 0.75 |

Note: Bold numbers indicate statistically significant group effect indicate by one-way ANOVAs.

Appendix 3. Post-hoc pairwise comparison of mean change in gait outcomes between arms

|  | Arm 2 (Ex+Cog) |  |  | Arm 3 (Ex+VitD) |  |  | Arm 4 (Ex) |  |  | Arm 5 (Control) |  |  |
| --- | --- | --- | --- | --- | --- | --- | --- | --- | --- | --- | --- | --- |
|  | MD (95% CI) | p | d | MD (95% CI) | p | d | MD (95% CI) | p | d | MD (95% CI) | p | d |
| **DTC gait speed counting backwards by 1s** |  |  |  |  |  |  |  |  |  |  |  |  |
| Arm 1 (Ex+Cog+VitD) | 2.78 (-4.68, 10.26) | 0.84 | 0.25 | -2.4(-9.82, 5.01) | 0.90 | -0.22 | -4.72 (-12.37, 2.92) | 0.43 | -0.43 | 0.09 (-7.76, 7.95) | 0.99 | 0 |
| Arm 2 (Ex+Cog) |  |  |  | -5.19(-12.55, 2.16) | 0.30 | -0.47 | -7.51 (-15.1, 0.08) | 0.05 | -0.68 | -2.69(-10.49, 5.10) | 0.88 | -0.24 |
| Arm 3 (Ex+VitD) |  |  |  |  |  |  | -2.31 (-9.85, 5.22) | 0.92 | -0.21 | 2.5 (-5.25, 10.25) | 0.90 | 0.22 |
| Arm 4 (Ex) |  |  |  |  |  |  |  |  |  | 4.81 (-3.15, 12.79) | 0.46 | 0.43 |
| **DTC gait speed naming animals** |  |  |  |  |  |  |  |  |  |  |  |  |
| Arm 1 (Ex+Cog+VitD) | 9.16 (-3.04, 21.36) | 0.24 | 0.51 | 1.31 (-10.8, 13.43) | 0.99 | 0.07 | 8.3 (-4.18, 20.79) | 0.36 | 0.46 | 8.24 (-4.59, 21.07) | 0.39 | 0.45 |
| Arm 2 (Ex+Cog) |  |  |  | -7.84 (-19.87, 4.18) | 0.38 | -0.43 | -0.85 (-13.25, 11.54) | 0.99 | -0.05 | -0.91 (-13.66, 11.82) | 0.99 | -0.05 |
| Arm 3 (Ex+VitD) |  |  |  |  |  |  | 6.99 (-5.32, 19.30) | 0.52 | 0.39 | 6.92 (-5.73, 19.58) | 0.56 | 0.38 |
| Arm 4 (Ex) |  |  |  |  |  |  |  |  |  | -0.06 (-13.08, 12.95) | 0.99 | 0 |
| **DTC gait speed serial 7s** |  |  |  |  |  |  |  |  |  |  |  |  |
| Arm 1 (Ex+Cog+VitD) | 1.76 (-7.99, 11.52) | 0.99 | 0.12 | -2.67 (-12.36, 7.01) | 0.94 | -0.19 | 3.92 (-6.06, 13.91) | 0.82 | 0.27 | 2.98 (-7.27, 13.24) | 0.93 | 0.2 |
| Arm 2 (Ex+Cog) |  |  |  | -4.44 (-14.05, 5.17) | 0.71 | -0.31 | 2.15 (-7.76, 12.07) | 0.98 | 0.15 | 1.22 (-8.97, 11.41) | 0.99 | 0.08 |
| Arm 3 (Ex+VitD) |  |  |  |  |  |  | 6.59 (-3.25, 16.44) | 0.35 | 0.46 | 5.66 (-4.46, 15.78) | 0.54 | 0.39 |
| Arm 4 (Ex) |  |  |  |  |  |  |  |  |  | -0.93 (-11.34, 9.47) | 0.99 | -0.06 |
| **Gait stride time variability** |  |  |  |  |  |  |  |  |  |  |  |  |
| Arm 1 (Ex+Cog+VitD) | -0.98 (-2.64, 0.66) | 0.47 | -0.40 | -0.21 (-1.85, 1.42) | 0.99 | -0.09 | -0.5 (-2.19, 1.18) | 0.92 | -0.21 | -0.8 (-2.54, 0.93) | 0.7 | -0.33 |
| Arm 2 (Ex+Cog) |  |  |  | 0.77 (-0.85, 2.40) | 0.68 | 0.32 | 0.48 (-1.19, 2.16) | 0.93 | 0.20 | 0.18 (-1.54, 1.9) | 0.99 | 0.07 |
| Arm 3 (Ex+VitD) |  |  |  |  |  |  | -0.29 (-1.96, 1.37) | 0.99 | -0.12 | -0.59 (-2.31, 1.12) | 0.87 | -0.24 |
| Arm 4 (Ex) |  |  |  |  |  |  |  |  |  | -0.30 (-2.06, 1.46) | 0.99 | -0.12 |
| **Stride time variability in fast gait** |  |  |  |  |  |  |  |  |  |  |  |  |
| Arm 1 (Ex+Cog+VitD) | 2.14 (-1.50, 5.79) | 0.49 | 0.40 | 1.73 (-1.88, 5.36) | 0.68 | 0.32 | 0.91 (-2.82, 4.64) | 0.96 | 0.17 | 0.31 (-3.52, 4.14) | 0.99 | 0.05 |
| Arm 2 (Ex+Cog) |  |  |  | -0.4 (-4.00, 3.19) | 0.99 | -0.08 | -1.23(-4.94to2.47) | 0.89 | -0.23 | -1.83 (-5.64, 1.97) | 0.67 | -0.33 |
| Arm 3 (Ex+VitD) |  |  |  |  |  |  | -0.82 (-4.51, 2.85) | 0.97 | -0.15 | -1.42 (-5.21, 2.36) | 0.83 | -0.26 |
| Arm 4 (Ex) |  |  |  |  |  |  |  |  |  | -0.59 (-4.49, 3.29) | 0.99 | -0.11 |
| **DTC stride time variability counting backwards by 1s** |  |  |  |  |  |  |  |  |  |  |  |  |
| Arm 1 (Ex+Cog+VitD) | -19.18 (-244.71, 206.34) | 0.99 | -0.06 | -52.46 (-276.39, 171.47) | 0.97 | -0.16 | 79.25 (-151.58, 310.09) | 0.88 | 0.24 | -126.62 (-363.75, 110.51) | 0.58 | -0.37 |
| Arm 2 (Ex+Cog) |  |  |  | -33.27 (-255.5, 188.96) | 0.99 | -0.10 | 98.44 (-130.74, 327.63) | 0.76 | 0.29 | -107.43 (-342.95, 128.09) | 0.71 | -0.32 |
| Arm 3 (Ex+VitD) |  |  |  |  |  |  | 131.71 (-95.9, 359.34) | 0.50 | 0.39 | -74.15 (-308.16, 159.84) | 0.9 | -0.22 |
| Arm 4 (Ex) |  |  |  |  |  |  |  |  |  | -205.87 (-446.49, 34.74) | 0.13 | -0.61 |
| **DTC stride time variability serial 7s** |  |  |  |  |  |  |  |  |  |  |  |  |
| Arm 1 (Ex+Cog+VitD) | 494.92 (-119.57, 1109.41) | 0.17 | 0.54 | 337.83(-272.32, 947.99) | 0.546 | 0.371 | 403.61(-225.36, 1032.58) | 0.39 | 0.44 | 75.74(-570.36, 721.85) | 0.99 | 0.08 |
| Arm 2 (Ex+Cog) |  |  |  | -157.08(-762.6, 448.43) | 0.953 | -0.17 | -91.31(-715.78, 533.16) | 0.99 | -0.1 | -419.17(-1060.91, 222.56) | 0.37 | -0.46 |
| Arm 3 (Ex+VitD) |  |  |  |  |  |  | 65.77(-554.43, 685.98) | 0.99 | 0.072 | -262.08(-899.67, 375.49) | 0.78 | -0.28 |
| Arm 4 (Ex) |  |  |  |  |  |  |  |  |  | -327.86(-983.47, 327.74) | 0.64 | -0.36 |
| **DTC stride time variability naming animals** |  |  |  |  |  |  |  |  |  |  |  |  |
| Arm 1 (Ex+Cog+VitD) | 43.62 (-103.9, 191.15) | 0.3 | 0.20 | -25.72 (-172.21, 120.76) | 0.99 | -0.12 | 57.62 (-93.38, 208.62) | 0.83 | 0.26 | 19.16 (-135.95, 174.28) | 0.99 | 0.08 |
| Arm 2 (Ex+Cog) |  |  |  | -69.35 (-214.72, 76.02) | 0.68 | -0.32 | 13.99 (-135.93, 163.91) | 0.99 | 0.06 | -24.46 (-178.53, 129.6) | 0.99 | -0.11 |
| Arm 3 (Ex+VitD) |  |  |  |  |  |  | 83.34 (-65.55, 232.24) | 0.54 | 0.38 | 44.88 (-108.18, 197.95) | 0.92 | 0.2 |
| Arm 4 (Ex) |  |  |  |  |  |  |  |  |  | -38.45 (-195.85, 118.94) | 0.96 | -0.17 |
| **Gait stride length variability** |  |  |  |  |  |  |  |  |  |  |  |  |
| Arm 1 (Ex+Cog+VitD) | -1.47 (-4.33, 1.38) | 0.61 | -0.35 | -1.15(-3.98, 1.68) | 0.80 | -0.27 | -1.59 (-4.52, 1.33) | 0.56 | -0.38 | -1.88 (-4.89, 1.11) | 0.41 | -0.44 |
| Arm 2 (Ex+Cog) |  |  |  | 0.32(-2.48, 3.14) | 0.99 | 0.08 | -0.11 (-3.02, 2.78) | 0.99 | -0.03 | -0.41(-3.39, 2.57) | 0.99 | -0.09 |
| Arm 3 (Ex+VitD) |  |  |  |  |  |  | -0.44 (-3.32, 2.44) | 0.99 | -0.11 | -0.73 (-3.7, 2.22) | 0.95 | -0.17 |
| Arm 4 (Ex) |  |  |  |  |  |  |  |  |  | -0.29 (-3.34, 2.75) | 0.99 | -0.06 |
| **Stride length variability in fast gait** |  |  |  |  |  |  |  |  |  |  |  |  |
| Arm 1 (Ex+Cog+VitD) | 0.73 (-0.93, 2.41) | 0.74 | 0.30 | 0.36 (-1.30, 2.02) | 0.98 | 0.146 | 0.9 (-0.81, 2.61) | 0.60 | 0.36 | -0.89 (-2.65, 0.87) | 0.63 | -0.35 |
| Arm 2 (Ex+Cog) |  |  |  | -0.37(-2.02, 1.27) | 0.97 | -0.15 | 0.16 (-1.53, 1.86) | 0.99 | 0.07 | -1.62 (-3.37, 0.12) | 0.08 | -0.65 |
| Arm 3 (Ex+VitD) |  |  |  |  |  |  | 0.53 (-1.15, 2.22) | 0.90 | 0.22 | -1.25(-2.98, 0.48) | 0.27 | -0.5 |
| Arm 4 (Ex) |  |  |  |  |  |  |  |  |  | -1.79(-3.57, -0.004) | **0.04** | -0.72 |
| **DTC stride length variability counting backwards by 1s** |  |  |  |  |  |  |  |  |  |  |  |  |
| Arm 1 (Ex+Cog+VitD) | 57.95 (-116.31, 232.21) | 0.89 | 0.22 | -89.19 (-262.23, 83.83) | 0.61 | -0.35 | 44.56 (-133.8, 222.93) | 0.96 | 0.172 | 20.33 (-162.9, 203.56) | 0.99 | 0.07 |
| Arm 2 (Ex+Cog) |  |  |  | -147.14 (-318.86, 24.57) | 0.13 | -0.57 | -13.38 (-190.48, 163.7) | 0.99 | -0.05 | -37.61 (-219.6, 144.37) | 0.97 | -0.14 |
| Arm 3 (Ex+VitD) |  |  |  |  |  |  | 133.76 (-42.12, 309.64) | 0.23 | 0.52 | 109.52 (-71.28, 290.33) | 0.45 | 0.42 |
| Arm 4 (Ex) |  |  |  |  |  |  |  |  |  | -24.23 (-210.15, 161.69) | 0.99 | -0.09 |
| **DTC stride length variability serial 7s** |  |  |  |  |  |  |  |  |  |  |  |  |
| Arm 1 (Ex+Cog+VitD) | 167.43 (-15.88, 350.75) | 0.09 | 0.62 | 0.56 (-181.46, 182.58) | 0.99 | 0.002 | 64.47 (-123.16, 252.1) | 0.88 | 0.24 | 10.29 (-182.45, 203.04) | 0.99 | 0.03 |
| Arm 2 (Ex+Cog) |  |  |  | -166.87 (-347.51, 13.76) | 0.09 | -0.61 | -102.96 (-289.25, 83.33) | 0.55 | -0.38 | -157.14 (-348.58, 34.3) | 0.16 | -0.57 |
| Arm 3 (Ex+VitD) |  |  |  |  |  |  | 63.91 (-121.11, 248.93) | 0.88 | 0.24 | 9.73 (-180.47, 199.93) | 0.99 | 0.03 |
| Arm 4 (Ex) |  |  |  |  |  |  |  |  |  | -54.18 (-249.76, 141.4) | 0.94 | -0.19 |
| **DTC stride length variability naming animals** |  |  |  |  |  |  |  |  |  |  |  |  |
| Arm 1 (Ex+Cog+VitD) | -16.68 (-105, 71.63) | 0.99 | -0.13 | 14.82 (-72.86, 102.52) | 0.99 | 0.11 | 10.75 (-79.64, 101.15) | 0.99 | 0.08 | -20.77 (-113.63, 72.08) | 0.97 | -0.15 |
| Arm 2 (Ex+Cog) |  |  |  | 31.51 (-55.51, 118.53) | 0.86 | 0.24 | 27.44 (-62.31, 117.19) | 0.92 | 0.21 | -4.08 (-96.31, 88.14) | 0.99 | -0.03 |
| Arm 3 (Ex+VitD) |  |  |  |  |  |  | -4.07 (-93.21, 85.06) | 0.99 | -0.03 | -35.59 (-127.23, 56.03) | 0.82 | -0.27 |
| Arm 4 (Ex) |  |  |  |  |  |  |  |  |  | -31.52 (-125.75, 62.69) | 0.88 | -0.24 |

Note: In bold all statistically significant effects at p<0.05.

CI, Confidence interval; d, effect size; SE, Standard error.

Marginal means and standard errors obtained from linear mixed models are reported for within-group differences. Between-group differences were assessed using the interaction between time x intervention arm. Lower scores indicate cognitive improvement.

Appendix 4. Effect of exercise (aerobic-resistance training) intervention with addition of cognitive training and vitamin D at 6-month on Dual-Task Cost (DTC)

|  | All Interventions vs control  Arms 1+2+3+4 vs. Arm 5(ref) | | | | Adding cognitive intervention to exercises  Arms 1+2 vs. Arm 3+4(ref) | | | | Adding vitamin D intervention  Arms 1+3 vs. Arm 2+4(ref) | | | | Multidomain intervention  Arm 1 vs. Arm 5(ref) | | | |
| --- | --- | --- | --- | --- | --- | --- | --- | --- | --- | --- | --- | --- | --- | --- | --- | --- |
|  | Mean change (SE) within group | Mean difference between groups (95%CI) | p | d | Mean change (SE) within group | Mean difference between groups (95%CI) | p | d | Mean change (SE) within group | Mean difference between groups (95%CI) | p | d | Mean change (SE) within group | Mean difference between groups (95%CI) | p | d |
| **DTC gait speed counting backwards by 1s** |  | 1.12 (-3.51, 5.74) | 0.881 | 0.1 |  | -4.91 (-8.62, 1.20) | **0.009** | -0.45 |  | 3.53 (7.50, -0.44) | 0.90 | 0.30 |  | 0.09 (-6.01, 6,20) | 0.79 | 0.01 |
| Comparison group | -1.05 (0.95) |  |  |  | -3.26 (1.65) |  |  |  | 0.23 (1.28) |  |  |  | 0.57 (1.77) |  |  |  |
| Reference group | -2.17 (2.31) |  |  |  | 0.40 (1.14) |  |  |  | -3.30 (1.57) |  |  |  | -0.66 (1.75) |  |  |  |
| **DTC gait speed naming animals** |  | 3.62 (-11.14, 3.90) | 0.65 | 0.20 |  | -0.05 (-6.09, 5.99) | 0.81 | -0.003 |  | 8.08 (-2.20, 13.95) | 0.13 | 0.47 |  | 8.24 (-2.50, 18.98) | 0.61 | 0.40 |
| Comparison group | -1.61 (1.52) |  |  |  | -1.63 (2.25) |  |  |  | 2.34 (2.06) |  |  |  | 3.02 (3.51) |  |  |  |
| Reference group | -5.22 (4.10) |  |  |  | -1.58 (2.06) |  |  |  | -5.73 (2.15) |  |  |  | -5.22 (4.10) |  |  |  |
| **DTC gait speed serial 7s** |  | 2.32 (-3.63, 8.28) | 0.51 | 0.16 |  | -0.47 (-5.55, 4.61) | 0.96 | -0.03 |  | 4.17 (-0.86, 9.20) | **0.01** | 0.28 |  | 2.99 (-3.78, 9.75) | 0.22 | 0.23 |
| Comparison group | -3.04 (1.28) |  |  |  | -3.28 (1.70) |  |  |  | -1.01 (1.54) |  |  |  | -2.38 (2.29) |  |  |  |
| Reference group | -5.37 (2.49) |  |  |  | -2.81 (1.93) |  |  |  | -5.18 (2.04) |  |  |  | -5.37 (2.49) |  |  |  |
| **Habitual stride time variability** |  | -0.38 (-1.39, 0.63) | 0.46 | -0.16 |  | 0.15 (-0.65, 096) | 0.71 | 0.06 |  | -0.65 (-1.45, 0.15) | 0.11 | -0.28 |  | -0.81 (-2.49, 0.87) | 0.34 | -0.25 |
| Comparison group | 0.18 (0.20) |  |  |  | 0.26 (0.34) |  |  |  | -0.14 (0.35) |  |  |  | -0.25 (0.61) |  |  |  |
| Reference group | 0.56 (0.56) |  |  |  | 0.11 (0.21) |  |  |  | 0.51 (0.18) |  |  |  | 0.56 (0.56) |  |  |  |
| **Stride time variability in fast gait** |  | -0.91 (-3.13, 1.32) | 0.40 | -0.17 |  | 0.26 (-1,21, 1.73) | 0.71 | 0.06 |  | 0.66 (-0.81, 2.13) | 0.35 | 0.16 |  | 0.31 (-3.52, 4.14) | 0.87 | 0.04 |
| Comparison group | 0.36 (0.37) |  |  |  | 0.49 (0.59) |  |  |  | 0.68 (0.61) |  |  |  | 1.58 (0.98) |  |  |  |
| Reference group | 1.27 (1.72) |  |  |  | 0.23 (0.46) |  |  |  | 0.02 (0.41) |  |  |  | 1.27 (1.72) |  |  |  |
| **DTC stride time variability counting backwards by 1s** |  | -126.38 (-263.58, 10.82) | 0.07 | -0.38 |  | 19.14 (-101.00, 139.29) | 0.75 | 0.06 |  | 54.76 (-65.08, 174.61) | 0.37 | 0.16 |  | -126.62 (-285.39, 32.15) | 0.12 | -0.41 |
| Comparison group | -68.67 (30.26) |  |  |  | -59.17 (30.77) |  |  |  | -41.91 (30.83) |  |  |  | -68.91 (61.22) |  |  |  |
| Reference group | 57.71 (46.99) |  |  |  | -78.32 (52.63) |  |  |  | -96.67 (52.2) |  |  |  | 57.71 (46.99) |  |  |  |
| **DTC stride time variability serial 7s** |  | -233.75 (-610.93, 143.43) | 0.22 | -0.25 |  | 117.58 (-116.95, 352.10) | 0.32 | 0.17 |  | 277.49 (-46.96, 508.01) | **0.02** | 0.41 |  | 75.75 (-669.85, 821.34) | 0.84 | 0.05 |
| Comparison group | 34.88 (59.27) |  |  |  | 93.23 (114.88) |  |  |  | 170.50 (110.21) |  |  |  | 344.38 (221.10) |  |  |  |
| Reference group | 268.64 (309.59) |  |  |  | -24.35 (25.83) |  |  |  | -106.99 (30.16) |  |  |  | 268.64 (309.59) |  |  |  |
| **DTC stride time variability naming animals** |  | 1.35 (-88.51, 91.21) | 0.98 | 0.006 |  | -8.72 (-89.57, 72.13) | 0.83 | -0.04 |  | 63.54 (-16.59, 143.68) | 0.12 | 0.27 |  | 19.16 (-58.73, 97.05) | 0.62 | 0.13 |
| Comparison group | -34.27 (20.36) |  |  |  | -38.60 (17.96) |  |  |  | -3.22 (19.93) |  |  |  | -16.46 (31.06) |  |  |  |
| Reference group | -35.62 (21.06) |  |  |  | -29.88 (36.92) |  |  |  | -66.76 (35.81) |  |  |  | -35.62 (21.06) |  |  |  |
| **stride length variability** |  | -0.84 (-2.58, 0.90) | 0.34 | -0.20 |  | -0.61 (-2.14, 0.92) | 0.42 | -0.14 |  | -0.94 (-2.46, 0.58) | 0.21 | -0.21 |  | -1.89 (-5.19, 1.41) | 0.24 | -0.29 |
| Comparison group | -0.24 (0.39) |  |  |  | -0.55 (0.72) |  |  |  | -0.70 (0.74) |  |  |  | -1.30 (1.44) |  |  |  |
| Reference group | 0.59 (0.57) |  |  |  | 0.06 (0.28) |  |  |  | 0.24 (0.16) |  |  |  | 0.59 (0.57) |  |  |  |
| **Stride length variability in fast gait** |  | -1.38 (-2.40, 0.37) | 0.73 | -0.56 |  | 0.24 (-0.37, 0.85) | 0.25 | 0.14 |  | 0.63 (0.03, 1.23) | **0.05** | 0.36 |  | -0.89 (-2.58, 0.80) | 0.52 | -0.27 |
| Comparison group | -0.002 (0.15) |  |  |  | 0.12 (0.19) |  |  |  | 0.31 (0.23) |  |  |  | 0.49 (0.26) |  |  |  |
| Reference group | 1.38 (0.86) |  |  |  | -0.12 (0.24) |  |  |  | -0.32 (0.19) |  |  |  | 1.38 (0.86) |  |  |  |
| **DTC stride length variability counting backwards by 1s** |  | 18.60 (-88.76, 125.97) | 0.73 | 0.07 |  | -55.78 (-152.02, 40.47) | 0.25 | -0.20 |  | 97.48 (2.21, 192.74) | **0.05** | 0.35 |  | 20.33 (-42.00, 82.67) | 0.52 | 0.17 |
| Comparison group | 13.38 (24.35) |  |  |  | -14.30 (13.40) |  |  |  | 61.02 (45.51) |  |  |  | -0.002 (0.15) |  |  |  |
| Reference group | -5.22 (24.57) |  |  |  | 41.48 (47.10) |  |  |  | -36.46 (12.60) |  |  |  | -5.22 (24.57) |  |  |  |
| **DTC stride length variability serial 7s** |  | -47.69 (-161.25, 65.88 | 0.41 | -0.17 |  | -54.39 (-156.02, 47.25) | 0.29 | -0.18 |  | -118.04 (-218.08, -18.00) | **0.02** | -0.41 |  | -10.29 (-89.84, 69.25) | 0.80 | -0.07 |
| Comparison group | -19.61 (25.7) |  |  |  | -46.60 (37.92) |  |  |  | -79.96 (37.33) |  |  |  | 28.08 (27.32) |  |  |  |
| Reference group | 28.08 (27.32) |  |  |  | 7.79 (34.61) |  |  |  | 38.08 (34.22) |  |  |  | 38.37 (28.33) |  |  |  |
| **DTC stride length variability naming animals** |  | -22.92 -76.38, 30.55) | 0.40 | -0.18 |  | 21.38 (-23.40, 66.16) | 0.35 | 0.16 |  | 11.23 (-33.67, 56.13) | 0.62 | 0.09 |  | 20.77 (-44.36, 85.90) | 0.52 | 0.16 |
| Comparison group | -35.32 (11.31) |  |  |  | -24.71 (16.98) |  |  |  | -29.58 (16.71) |  |  |  | -12.40 (24.36) |  |  |  |
| Reference group | -12.40 (24.36) |  |  |  | -46.09 (14.94) |  |  |  | -40.81 (15.40) |  |  |  | -33.18 (21.73) |  |  |  |

Note: In bold all statistically significant effects at p<0.05. CI, Confidence interval; DTC, Dual task gait cost; p, p-value; SE, Standard error.

Marginal means and standard errors obtained from linear mixed models are reported for within-group differences. Between-group differences were assessed using the interaction between time x intervention arm. Lower scores indicate cognitive improvement.

Appendix 5 – One-way ANOVAs comparing the mean change of the cognitive task results during the dual task conditions

| Variables | Time points | Arm 1  (Ex+Cog+VitD)  Mean(SD) | Arm 2  (Ex+Cog)  Mean(SD) | Arm 3  (Ex+VitD)  Mean(SD) | Arm 4  (Ex)  Mean(SD) | Arm 5  (Control)  Mean(SD) | F | p-value |
| --- | --- | --- | --- | --- | --- | --- | --- | --- |
| Counting backwards by 1s, total | T0 | 9.18 (2.63) | 9.21 (2.74) | 9.21 (2.18) | 9.29 (2.93) | 8.77 (1.75) | - | - |
|  | T6 | 8.39 (2.50) | 9.32 (3.19) | 8.93 (1.90) | 8.79 (2.55) | 8.35 (2.71) | - | - |
|  | Mean Change(Δ) | -0.78 (0.49) | 0.10 (0.62) | -0.28(0.27) | -0.50 (0.49) | -0.41 (0.67) | 0.44 | 0.77 |
| Counting backwards by 1s, errors | T0 | 0.18 (0.48) | 0.04 (0.19) | 0.14 (0.53) | 0.04 (0.20) | 0.06 (0.24) | - | - |
|  | T6 | 0.14 (0.45) | 0.18 (0.39) | 0.07 (0.26) | 0 (0) | 0.06 (0.24) | - | - |
|  | Mean Change(Δ) | -0.03 (0.09) | 0.14 (0.08) | -0.07 (0.11) | -0.04 (0.04) | 0(0) | 1.02 | 0.39 |
| Serial 7s subtractions, total | T0 | 3.56 (1.47) | 3.65 (1.88) | 3.44 (1.19) | 3.39 (1.37) | 3.88 (2.13) | - | - |
|  | T6 | 3.40 (1.58) | 3.82 (1.27) | 3.64 (1.50) | 3.48 (1.44) | 3.56(1.55) | - | - |
|  | Mean Change(Δ) | -0.16(0.33) | 0.23 (0.39) | 0.20 (0.38) | 0.08 (0.39) | -0.31 (0.47) | 0.32 | 0.86 |
| Serial 7s subtractions, errors | T0 | 0.64 (0.91) | 1.04 (1.37) | 0.48 (0.71) | 0.65 (0.89) | 0.88 (1.20) | - | - |
|  | T6 | 0.60 (0.87) | 1.04 (1.22) | 0.92 (1.15) | 0.83(0.98) | 0.75 (1) | - | - |
|  | Mean Change(Δ) | -0.04 (0.21) | 0.03(0.21) | 0.44 (0.22) | 0.17 (0.26) | -0.12 (0.3) | 0.83 | 0.50 |
| Animals named, total | T0 | 6.11 (1.62) | 6.48 (1.42) | 5.86 (1.92) | 5.96 (1.49) | 5.56 (2.07) | - | - |
|  | T6 | 5.61 (1.29) | 5.93 (1.49) | 5.89 (1.65) | 5.30 (2.32) | 6.13 (1.75) | - | - |
|  | Mean Change(Δ) | -0.50 (0.43) | -0.55 (0.25) | -0.03 (0.40) | -0.65 (0.59) | 0.56 (0.57) | 1.00 | 0.40 |

Appendix 6 – Effect of exercise (aerobic-resistance training) intervention with addition of cognitive training and vitamin D at 6-month end point in the cognitive tasks

|  | Exercise Intervention  Arms 1+2+3+4 vs. Arm 5(ref) | | | | Adding cognitive intervention  Arms 1+2 vs. Arm 3+4(ref) | | | | Adding vitamin D intervention  Arms 1+3 vs. Arm 2+4(ref) | | | | Multidomain intervention  Arm 1 vs. Arm 5(ref) | | | |
| --- | --- | --- | --- | --- | --- | --- | --- | --- | --- | --- | --- | --- | --- | --- | --- | --- |
|  | Mean change (SE) within group | Mean difference between groups (95%CI) | P value | d | Mean change (SE) within group | Mean difference between groups (95%CI) | P value | d | Mean change (SE) within group | Mean difference between groups (95%CI) | P value | d | Mean change (SE) within group | Mean difference between groups (95%CI) | P value | d |
| **Counting backwards by 1s total said** |  | 0.05 (-1.27, 1.37) | 0.93 | 0.02 |  | -0.05 (-1.01, 0.92) | 0.89 | -0.02 |  | 0.36 (-0.60, 1.33) | 0.49 | 0.14 |  | 0.37 (-2.04, 1.29) | 0.70 | 0.14 |
| Comparison group | -0.36 (0.24) |  |  |  | -0.39 (0.27) |  |  |  | -0.17 (0.40) |  |  |  | -0.41 (0.68) |  |  |  |
| Reference group | -0.41 (0.68) |  |  |  | -0.34 (0.40) |  |  |  | -0.54 (0.28) |  |  |  | -0.78 (0.68) |  |  |  |
| **Serial 7s subtractions total** |  | 0.40 (-0.59, 1.40) | 0.45 | 0.22 |  | 0.11 (-0.64, 0.85) | 0.69 | 0.06 |  | 0.14 (-0.60, 0.89) | 0.72 | 0.08 |  | -0.15 (1.28, 0.98) | 0.83 | -0.09 |
| Comparison group | 0.09 (0.19) |  |  |  | 0.15 (0.27) |  |  |  | 0.16 (0.28) |  |  |  | -0.31 (0.47) |  |  |  |
| Reference group | -0.31 (0.47) |  |  |  | 0.04 (0.26) |  |  |  | 0.02 (0.25) |  |  |  | -0.16 (0.47) |  |  |  |
| **Naming animal total said** |  | -0.99 (-2.15, 0.17) | 0.10 | -0.45 |  | 0.2 (-0.64, 1.05) | 0.60 | 0.10 |  | -0.33 (-1.17, 0.52) | 0.38 | -0.15 |  | 1.06 (-0.38, 2.51) | 0.16 | 0.47 |
| Comparison group | -0.43 (0.21) |  |  |  | -0.32 (0.35) |  |  |  | -0.60 (0.30) |  |  |  | 0.56 (0.57) |  |  |  |
| Reference group | 0.56 (0.57) |  |  |  | -0.53 (0.25) |  |  |  | -0.27 (0.30) |  |  |  | -0.5 (0.57) |  |  |  |
| **Counting backwards total errors** |  | 0.28 (-0.89, 0.34) | 0.38 | 0.24 |  | 0.31 (-0.14, 0.76) | 0.16 | 0.28 |  | -0.10 (-0.55, 0.36) | 0.64 | -0.09 |  | -0.09 (-0.81, 0.64) | 0.38 | -0.08 |
| Comparison group | 0.15 (0.11) |  |  |  | 0.31 (0.17) |  |  |  | 0.10 (0.17) |  |  |  | -0.13 (0.30) |  |  |  |
| Reference group | -0.13 (0.30) |  |  |  | 0 (0.15) |  |  |  | 0.20 (0.16) |  |  |  | -0.04 (0.21) |  |  |  |
| **Serial 7s subtractions total errors** |  | 0 (-0.23, 0.23) | 0.45 | 0 |  | -0.11 (-0.29, 0.07) | 0.22 | -0.24 |  | 0.11 (-0.07, 0.29) | 0.22 | 0.24 |  | 0.04 (-0.21, 0.29) | 0.78 | -0.09 |
| Comparison group | 0 (0.05) |  |  |  | -0.06 (0.06) |  |  |  | 0.06 (0.05) |  |  |  | 0(0) |  |  |  |
| Reference group | 0 (0) |  |  |  | 0.05 (0.07) |  |  |  | -0.05 (0.07) |  |  |  | -0.04 (0.10) |  |  |  |

Note: In bold all statistically significant effects at p<0.05.

CI, Confidence interval; d, effect size; DTC, Dual task gait cost; p-value; SE, Standard error.

Marginal means and standard errors obtained from linear mixed models are reported for within-group differences. Between-group differences were assessed using the interaction between time x intervention arm. Lower scores indicate cognitive improvement.

Appendix Table 7 – Falls distribution across arms at baseline (T0), month 6 (T6), and month 12 (T12)

|  | Arm 1  (Ex+Cog+VitD) | | | Arm 2  (Ex+Cog) | | | Arm 3  (Ex+VitD) | | | Arm 4  (Ex) | | | Arm 5  (Control) | | |
| --- | --- | --- | --- | --- | --- | --- | --- | --- | --- | --- | --- | --- | --- | --- | --- |
|  | T0 | T6 | T12 | T0 | T6 | T12 | T0 | T6 | T12 | T0 | T6 | T12 | T0 | T6 | T12 |
|  | N (%) | N (%) | N (%) | N (%) | N (%) | N (%) | N (%) | N (%) | N (%) | N (%) | N (%) | N (%) | N (%) | N (%) | N (%) |
| **Falls** |  |  |  |  |  |  |  |  |  |  |  |  |  |  |  |
| 0 | 24 | 27 | 31 | 29 | 30 | 29 | 26 | 29 | 27 | 19 | 17 | 24 | 15 | 15 | 20 |
| 1 | 3 (8.8) | 5 (14.7) | 1 (3) | 5 (14.7) | 2 (5.8) | 4 (11.7) | 6 (16.6) | 5 (13.8) | 5 (14.2) | 6 (20) | 7 (23.3) | 3 (9.6) | 3 (10.7) | 4 (14.2) | 5 (17.8) |
| 2 | 4 (11.7) | 2 (5.8) | 1 (3) | 0 | 2 (5.8) | 0 | 4 (11.1) | 2 (5.5) | 1 (2.9) | 0 | 2 (5.5) | 4 (12.9) | 0 | 6 (21.4) | 1 (3.5) |
| 3 | 3 (8.8) | 0 | 0 | 0 | 0 | 1 (2.9) | 0 | 0 | 1 (2.9) | 0 | 0 | 0 | 3 (10.7) | 3 (10.7) | 1 (3.5) |
| ≥4 | 0 | 0 | 0 | 0 | 0 | 0 | 0 | 0 | 0 | 5 (16.6) | 4 (13.3) | 0 | 7 (25) | 0 | 1 (3.5) |
| **Injurious falls** |  |  |  |  |  |  |  |  |  |  |  |  |  |  |  |
| 0 | 30 | 22 | 32 | 31 | 33 | 33 | 31 | 33 | 32 | 24 | 25 | 31 | 25 | 22 | 25 |
| 1 | 4 (11.7) | 2 (5.8) | 1 (3) | 3(8.8) | 1 (2.9) | 1 (2.9) | 5 (13.8) | 3 (8.3) | 1 (2.9) | 6 (20) | 5 (16.6) | 0 | 3 (10.7) | 6 (21.4) | 3 (10.7) |
| 2 | 0 | 0 | 0 | 0 | 0 | 0 | 0 | 0 | 0 | 0 | 0 | 0 | 0 | 0 | 0 |
| 3 | 0 | 0 | 0 | 0 | 0 | 0 | 0 | 0 | 0 | 0 | 0 | 0 | 0 | 0 | 0 |
| ≥4 | 0 | 0 | 0 | 0 | 0 | 0 | 0 | 0 | 0 | 0 | 0 | 0 | 0 | 0 | 0 |

Appendix 8 – Effect of aerobic-resistance exercise intervention arms on falls rate at 6-month

|  | Falls |  | Injurious falls | |
| --- | --- | --- | --- | --- |
|  | IRR (95%CI) | p | IRR (95%CI) | p |
|  |  |  |  |  |
| Arm 1 (Ex+Cog+VitD) | 1.08 (0.46, 2.59) | 0.84 | 0.77 (0.23, 2.44) | 0.66 |
| Arm 5 (control) | Reference |  | Reference |  |
|  |  |  |  |  |
| Arm 2 (Ex+Cog) | 0.34 (0.08, 1.10) | 0.09 | 0.13 (0.01, 0.74) | 0.06 |
| Arm 5 (control) | Reference |  | Reference |  |
|  |  |  |  |  |
| Arm 3 (Ex+VitD) | 0.55 (0.18, 1.51) | 0.26 | 0.27 (0.04, 1.11) | 0.10 |
| Arm 5 (control) | Reference |  | Reference |  |
|  |  |  |  |  |
| Arm 4 (Ex) | 0.53 (0.17, 1.46) | 0.23 | 0.38 (0.08, 1.38) | 0.16 |
| Arm 5 (control) | Reference |  | Reference |  |

Note: IRR, Incident rate ratio; CI, Confidence interval

Appendix 9 – The mean Physical Activity Scale for the Elderly (PASE) score at baseline, 6-month, and 12-month endpoints across arms

|  | Baseline  mean (SD) | 6-month  mean (SD) | 12-month  mean (SD) | p-value |
| --- | --- | --- | --- | --- |
| Arm 1 (Ex+Cog+VitD) | 114 (57.2) | 141 (66.7) | 118 (56.5) | 0.03 |
| Arm 2 (Ex+Cog) | 122 (67.0) | 123 (63.4) | 125 (64.2) | 0.85 |
| Arm 3 (Ex+VitD) | 117 (62.1) | 114 (53.3) | 114 (49.2) | 0.83 |
| Arm 4 (Ex) | 99.8 (58.9) | 114 (63.0) | 93.5 (53.5) | 0.22 |
| Arm 5 (control) | 104 (54.8) | 98.2 (41.0) | 103 (58.4) | 0.81 |
| Overall | 111 (60.1) | 118 (59.2) | 111 (56.7) | 0.33 |
